# Transcription factors in eukaryotic cells can functionally regulate gene expression by acting in oligomeric assemblies formed from an intrinsically disordered protein phase transition enabled by molecular crowding

**DOI:** 10.1080/21541264.2018.1475806

**Published:** 2018-08-09

**Authors:** Mark C. Leake

**Affiliations:** Departments of Physics and Biology, Biological Physical Sciences Institute, University of York, York, UK

**Keywords:** Gene expression, transcription factors, single-molecule, super-resolution, cell signaling, intrinsically disordered protein, phase transition, molecular crowding, fluorescent protein

## Abstract

High-speed single-molecule fluorescence microscopy in vivo shows that transcription factors in eukaryotes can act in oligomeric clusters mediated by molecular crowding and intrinsically disordered protein. This finding impacts on the longstanding puzzle of how transcription factors find their gene targets so efficiently in the complex, heterogeneous environment of the cell.

**Abbreviations** CDF - cumulative distribution function; FRAP - fluorescence recovery after photobleaching; GFP - Green fluorescent protein; STORM - stochastic optical reconstruction microscopy; TF - Transcription factor; YFP - Yellow fluorescent protein

## Introduction

Cells regulate gene expression through binding of transcription factors (TFs) to promoters to turn gene expression on or off [1,2]. Simulations show that the time it takes for TFs to find their targets through pure 3D diffusion alone is ~six orders of magnitude larger than what is observed experimentally [3]. Hypotheses to explain this observation have included TF heterogeneous mobility comprising a combination of free 3D diffusion combined with sliding and hopping on the DNA plus longer jumps between different DNA strands called intersegment transfer [4–6]. In eukaryotic cells, TF localization fluctuates, often between cytoplasm and nucleus [7]. Although it has been observed that promoters can pool on the genome in clusters [8] it has not previously been seen that TFs themselves act in clusters, but instead are largely assumed to act as single molecules. Simulations which embody diffusion and binding suggest that multivalent TFs could, in principle, facilitate intersegment transfer [9]. Previously, single-molecule fluorescence microscopy has been used to study TF localization in living cells across a range of model organisms, including bacteria, yeast and multi-cellular organisms [10–16]. Many studies suggest complexities in diffusion and binding [4,11,12,15,17] which may include intersegmental transfer [4,11,17]. However, until now, the direct experimental evidence for intersegmental transfer has been limited.

Many of the important features of gene expression control in eukaryotes are exemplified in the model unicellular microorganism *Saccharomyces cerevisiae* (budding yeast). In particular, its glucose sensing pathway presents an experimentally tractable system to study gene regulation. Here, control of gene expression is achieved by TFs which include the Zn finger DNA binding protein Mig1 [18] that acts to repress expression from targets including *GAL* genes involved in glucose metabolism [19]. Mig1 localizes towards the nucleus if the extracellular glucose concentration is increased [20], correlated to its own dephosphorylation by a protein called Snf1 [21,22].

In recent investigations from my own group [23] the spatiotemporal dynamics and kinetics of gene regulation in live *S. cerevisiae* cells, using its glucose sensing pathway as a model for signal transduction, was explored using physics methods which enable the understanding of the processes of life one molecule at a time [24,25], employing ‘single-molecule optical proteomics’ tools [26]. The combination of these advanced light microscopy with genetics techniques has previously enabled valuable insights into the activities of several other processes for low copy number proteins [27] in both unicellular organisms and single cells from more complex multicellular organisms [28]. These single-molecule/cell and super-resolution microscopy tools have in particular been applied to integrated membrane proteins [29,30], such as interaction networks like oxidative phosphorylation [31–35], cell division processes [36–38] and protein translocation [39], along with bacterial cell motility [40–43]. The tools can also probe the aqueous environment of cells as opposed to just on their hydrophobic cell membrane surface, including processes of DNA replication/remodeling/repair [44–46], and systems more directly relevant to biomedicine such as bacterial infection [47–49].

In this Points of View article I discuss further the findings from my team from single-molecule fluorescence microscopy to track functional TFs with very high speed to match typical rates of protein diffusion in live cells and thereby enable “blur-free” observations. We were able to quantify the composition and dynamics of Mig1 under normal and perturbed conditions which affected its state of phosphorylation, and also performed experiments on a protein called Msn2 which functions antagonistically, i.e. instead as an enhancer/activator, for many of the same Mig1 target genes [50] through a completely different signaling pathway. The results showed unexpectedly that Mig1 binds to its target genes as an oligomeric cluster which has stoichiometries in the range ~6–9 molecules. We found evidence that Mig1 molecules in a cluster are glued together through interactions of intrinsically disordered peptide sequences innervated by molecular crowding depletion forces in the cell. Our findings may reveal a more general eukaryotic cell strategy for the control of gene expression which uses intrinsic disorder of many TFs to form clusters that then enable large reductions in the time taken to find a given target gene.

## Results

### Single-molecule optical proteomics indicates the presence of Mig1 oligomeric clusters

**Figure 1.**
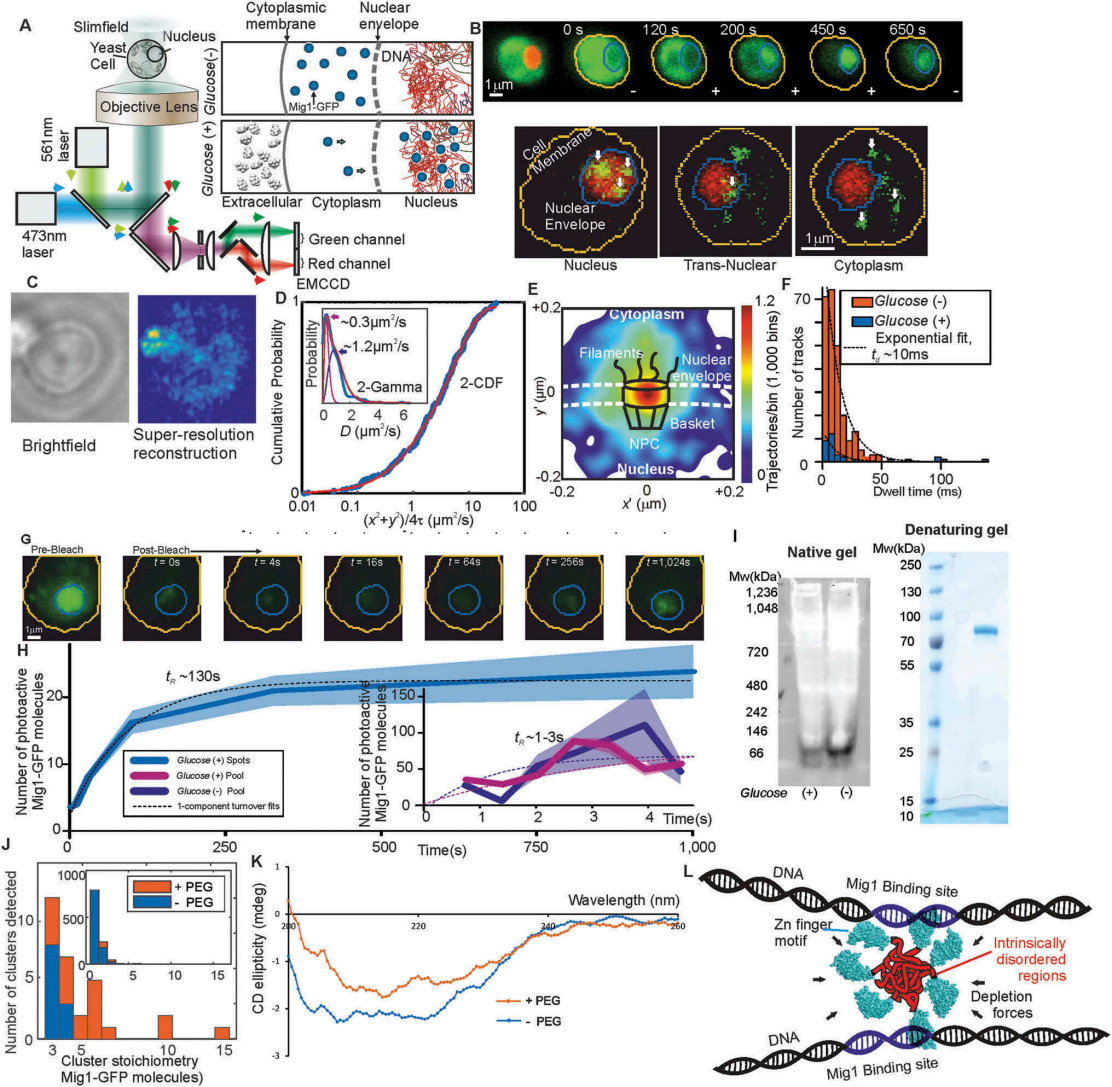
TFs form clusters in eukaryotic cell. (a) Schematic of millisecond Slimfield microscopy. (b) Fluorescence imaging of Mig1-GFP (green) with nucleus indicated (red) by Nrd1-mCherry, showing different cellular locations, stoichiometry determined by step-wise photobleaching that can be measured using Fourier analysis and edge-detection filters [52,79,80]. (c) STORM imaging using Mig1-mEos2. (d) Mobility analysis for cumulative distribution function (CDF) and Gamma fits. (e) Mig-GFP localization through a nuclear pore complex. (f) Dwell time for tracks translocating the nuclear envelope. (g) Images and (h) analysis for FRAP indicating turnover of nuclear Mig1-GFP. (i) Native and denaturing gels on purified Mig1-GFP. (j) Mig1-GFP cluster stoichiometry in presence/absence of molecular crowding. (k) Circular dichroism spectra in presence/absence of molecular crowding. (l) Cartoon model for shape of a Mig1 cluster in vicinity of DNA strands.

We used millisecond Slimfield single-molecule fluorescence imaging [44,45,51] on live *S. cerevisiae* cells (Figure 1(a)) using a green fluorescent protein (GFP) reporter for Mig1 integrated into the genome, including mCherry reporter on the RNA polymerase subunit protein Nrd1 to indicate the position of the cell nucleus. Slimfield was optimized for single-molecule detection sensitivity by using an *in vitro* imaging assay [52]. We also measured the maturation effect of the fluorescent proteins in these cells [53] and estimate in to be <15% immature fluorescent protein over the timescale of imaging experiments. Note, Slimfield limits the observation area to an equivalent diameter of <10 μm in the lateral plane to achieve rapid imaging sample times of millisecond and, in some instancesm sub-millisecond levels [54], but is less ideal to eukaryotic imaging of cells with larger nuclei. A host of other single-molecule techniques based on light-sheet imaging have larger fields of view, and also combine low background and low light toxicity. For the interested reader, these include: HILO (by Tokunaga M.N. et al. [55]. AFM cantilever lightsheet (by Gebhardt, J.C. et al. [11]), lattice light-sheet (by Chen B.C. et al. [56]), multi-focus (by Abrahamsson S. et al [57].), remote focusing (by Yang et al [58].), and diagonally scanned light sheet (by Dean et al [59].).

Under depleted/elevated extracellular *glucose* (-/+) we measured cytoplasmic and nuclear Mig1 localization bias respectively, visible in individual cells by our generating rapid microfluidic exchange (a few seconds) of extracellular fluid (Figure 1(b)), and resolved two components under both conditions consistent with a diffuse monomeric pool and distinct oligomeric foci of Mig1 (Figure 1(c)). The foci were also visible as hotspots using the green-red photoswitchable fluorescent protein mEos2 [60] excited by super-resolution stochastic optical reconstruction microscopy (STORM) (Figure 1(c)), with modeling using 3C structural data of the yeast chromosome [61] and sequence alignment analysis for the location of Mig1 target promoters supporting the hypothesis that the majority of Mig1 clusters were specifically binding to Mig1 target genes.

Nanoscale tracking determined the position of tracked Mig1 foci to a lateral precision of 40 nm [32,62] coupled to stoichiometry analysis using stepwise photobleaching of GFP [52] and single cell copy number analysis [63]. An additional output from the tracking was the effective diffusion coefficient *D* as a function of its location in either the cytoplasm, nucleus or translocating across the nuclear envelope, as well as the copy number of Mig1 molecules associated with each subcellular region and in each cell as a whole, indicating ~850–1,300 Mig1 molecules per cell dependent on extracellular glucose. It should be noted that confinement may affect the apparent diffusion coefficient in the small volume of a yeast nucleus if the length the mean square displacement (MSD) of tracked particles is comparable to the diameter of the nucleus, however, if our case only the short length scale MSD regions are considered to determine *D*.

In control experiments, a modified strain [50] generated with a binding site for protein PP7 on mRNA produced by one of the Mig1 target genes called *GSY1* showed colocalization between PP7-GFP expressed off a plasmid and Mig1-mCherry expressed genomically under high glucose conditions. We also observed similar clustering and co-localization to PP7 for the antagonistic TF Msn2. These PP7 co-localization results suggest that clusters both of Mig1 and Msn2 are *functionally* active in regulating target gene expression of the test target gene *GSY1*.

### Cytoplasmic Mig1 diffuses rapidly but nuclear Mig1 can be mobile and immobile

Cytoplasmic Mig1 fluorescent foci at *glucose* (±), and nuclear foci at *glucose* (-), were consistent with just a single mobile population whose *D* of 1–2 μm^2^/s consistent with earlier observations. However, nuclear foci at *glucose* (+) indicated a mixture of mobile and immobile components (Figure 1(d)). These results suggested 20–30% of nuclear foci are immobile, consistent with a DNA-bound state. MSD analysis of foci tracks indicated Brownian diffusion over a few tens of ms but increasingly anomalous diffusion over longer timescales, consistent with *glucose* (+) Mig1 diffusion being impacted by interactions with nuclear structures, similar to that reported for other TFs [64]. Here, this interaction depended on extracellular glucose despite Mig1 requiring a pathway of proteins to detect it, unlike the more direct detection mechanism of the prokaryotic *lac* repressor. Control experiments with Zn finger deletion strains of Mig1 indicated that Mig1 clusters bind to the DNA via their Zn finger motif with direct glucose dependence. At the high laser exceition intensities used for Slimfiled imaging photobleaching is rapid, and so typically a single GFP molecule will photobleach on average after 5–10 consecutive image frame. To account for this we interpolate observed foci brightness values back to the start of each photobleach using an exponential photobleach function. We observed no direct evidence for irreversible photobleaching (i.e. “photoblinking”) with GFP at these intensities, though other fluorescent proteins such as YFP have been known to exhibit such blinking behavior, which if so would need to be further characterized, for example using surface imnmobilized purified YFP *in vitro* samples. A general compromise here, however, is to confine tracking analysis to typically less than 100 ms of laser exposure so that irreversible photoblinking is more dominant than reversible blinking.

### Mig1 nuclear pore complex selectivity is mediated by interactions distant from the nuclear envelope

We compared the spatiotemporal dynamics of different Mig1 clusters during translocation by converting trans-nuclear tracks into coordinates parallel and perpendicular to the measured nuclear envelope location, and synchronizing coordinate origins to be at the nuclear envelope crossing point for a given foci track. A heat map of spatial locations of translocating clusters indicated a hotspot of comparable volume to the nuclear pore complexes and accessory structures [65,66] (Figure 1(e)). The dwell time during nuclear envelope translocation was ~10 ms, similar to previous estimates for transport factors [67] but here found to be insensitive to glucose (Figure 1(f)), demonstrating that there is no direct selectivity on the basis of TF phosphorylation state by nuclear pore complexes themselves which suggests that cargo selectivity mechanisms of nuclear transport [68] are blind to phosphorylation state. Coupled with the observation that Mig1 at *glucose* (-) does not exhibit immobility in the nucleus and that Mig1 lacking the Zn finger still accumulates in the nucleus at *glucose* (+) this suggests that Mig1 localization is driven by changes in Mig1 binding affinity to other proteins, e.g. the general co-repressor complex at the genome [69], or outside the nucleus not involving the nuclear pore complex.

### Mig1 nuclear clusters turn over in >100 s

By modifying the microscope we were able to implement fluorescence recovery after photobleaching (FRAP) to probe nuclear turnover of Mig1, by focusing a separate laser onto just the nucleus, photobleaching this region with a rapid 200 ms pulse, and quantifying any subsequent fluorescence intensity recovery into that region (Figure 1(g)). We could then acquire images with millisecond precision for individual frames but stroboscopically illuminating to extend the range of time scales for recovery before significant GFP photobleaching occurred, enabling FRAP observations at a single-molecule precision to timescales >1,000 s. Analyzes demonstrated measurable recovery for both foci and the diffuse pool components in the nucleus, which could be fitted by single exponential functions indicating fast recovery of pool at both *glucose* (-) and (+) with a time constant of just a few seconds but a larger time constant at *glucose* (+) for nuclear foci of at least ~100s (Figure 1(h)), with recovery of intensity being consistent with units of ~7–9 GFP molecules for the foci component but no obvious periodicity in stoichiometry measurable from pool recovery. These data suggested that molecular turnover at nuclear foci of Mig1 bound to target genes occurred in units of whole Mig1 clusters.

### Clusters are stabilized by molecular crowding and intrinsic disorder

Native, denaturing gel electrophoresis and western blots on purified extracts from Mig1-GFP cells (Figure 1(i)) indicated a single band corresponding to Mig1. *In vitro* Slimfield imaging of purified Mig1-GFP under identical imaging conditions for live cells similarly indicated monomeric Mig1-GFP foci in addition to a small fraction of brighter foci which were consistent with chance overlap of monomer GFP images. However, addition of a molecular crowding reagent in the form of low molecular weight polyethylene glycol (PEG) at a concentration known to correspond to small molecule “depletion” forces in cells [70] resulted in significant numbers of oligomers (Figure 1(j)), suggesting that Mig1 clusters present in live cells regardless of glucose may be stabilized by depletion components that are lost during biochemical purification.

Secondary structure predictions suggested significant regions of disorder away from the Zn finger binding motif. We measured changes in circular dichroism of the Mig1 fusion construct upon addition of PEG (Figure 1(k)) in a wavelength range known to be sensitive to transitions between ordered and intrinsically disordered states [71,72]. We also noted similar levels of disorder content in the Msn2 protein far from the Zn finger motif. These observations suggested a TF “molecular bipolarity”, in regards to disorder content, which stabilizes a cluster compact core focused around the disordered regions that undergo a putative phase transition to a more structure state, while exposing Zn fingers and positive surface charges to enable specific and non-specific interactions with accessible DNA strands (Figure 1(l)).

### Perspective

Our findings address aspects of functional gene regulation in live cells which have hitherto been unexplored, using biophysical technology that has not been available until recently. The results strongly support a functional link between Mig1 and Msn2 TF clusters and target gene expression; a biological role of multivalent TFs for enhancing intersegmental transfer had been elucidated previously in simulations [9] but unobserved experimentally until our discoveries here, and so our findings impact on the longstanding question of how TFs might find their targets in the genome so efficiently. Clustering of a range of nuclear factors has been observed previously using single-molecule techniques, such as transient RNA Polymerase II cluster dynamics in living cells using time-correlated PALM (tc-PALM) [73,74]. Also functional nuclear protein clusters have been seen [75] and the Bicoid transcription factor in fruit fly embryos has been observed to form clusters mediated in part mediated by intrinsically disordered peptide sequences [76].

Quantifying nearest-neighbor distances between Mig1 promoter sites in the *S. cerevisiae* genome from the 3C model indicates 20–30% are <50 nm apart, small enough to enable different DNA segments to be linked though intersegment transfer by a single cluster [6,9], which would also enable in principle simultaneous binding of >1 gene target from just a single TF cluster. There is a net positive charge in the vicinity of Zn finger motifis, and this would also enable non-specific electrostatic interactions with the negatively charged phosphate backbone of DNA, facilitating 1D sliding diffusion of the protein along a DNA strand. Thus, a cluster may be able to slide along DNA in a largely sequence-independent manner and undergo intersegmental transfer to a neighboring strand relatively easily, either spontaneously or stimulated by the presence of protein barriers on the DNA in a process likely to have some sequence dependence when an obstacle is encountered. In particular, bound RNA polymerases present during gene transcription at sequence specific sites could act as roadblocks to kick off translocating clusters from a DNA strand, to again facilitate intersegmental transfer and thus increase the ultimate chances that TF clusters will encounter one of the gene targets and specifically bind via the Zn finger motif, thus predominantly circumventing the requirement for significant amounts of slow 3D diffusion in the nucleoplasm.

Our discovery is, to our knowledge, the first to make a link between predicted disorder and the ability to form oligomeric clusters in TFs. Our findings may potentially offer some insights into addressing the longstanding question of why in general there is so much predicted disorder in eukaryote transcription factors; ~90% of eukaryotic TFs indicate significant proportions of sequences with disordered content [77]. Our finding that protein interactions based on relatively weak molecular crowding depletion forces has functional relevance in several areas of cell biology, such as processes involving aggregation mediated through intrinsic disorder interactions; for example, those of amyloid plaques found in neurodegenerative disorders including Alzheimer’s and Parkinson’s diseases [78]. Increased understanding of the clustering mechanism might therefore be of value in understanding the progression of these diseases. Open questions remain though: for example, are clusters homo-oligomeric or do they contain multiple different TFs? How is specificity maintained inside a cluster? Are the components of the clusters themselves dynamic and undergo molecular turnover? Can the ability to cluster be controlled, for example by switching the state of phosphorylation?
